# Increased PHGDH expression promotes aberrant melanin accumulation

**DOI:** 10.1186/s12885-019-5933-5

**Published:** 2019-07-22

**Authors:** Katherine R. Mattaini, Mark R. Sullivan, Allison N. Lau, Brian P. Fiske, Roderick T. Bronson, Matthew G. Vander Heiden

**Affiliations:** 1 Koch Institute for Integrative Cancer Research, Cambridge, 02139 MA USA; 2 Department of Biology, Massachusetts Institute of Technology, Cambridge, MA 02139 USA; 3 Rodent Histopathology Core, Harvard Medical School, Boston, Cambridge, MA 02111 USA; 4 Dana-Farber Cancer Institute, Boston, MA 02215 USA; 5 Broad Institute, Cambridge, MA 02139 USA

## Abstract

**Background:**

Copy number gain of the D-3-phosphoglycerate dehydrogenase (*PHGDH*) gene, which encodes the first enzyme in serine biosynthesis, is found in some human cancers including a subset of melanomas.

**Methods:**

In order to study the effect of increased *PHGDH* expression in tissues in vivo, we generated mice harboring a *PHGDH*^*tetO*^ allele that allows tissue-specific, doxycycline-inducible PHGDH expression, and we analyzed the phenotype of mice with a ubiquitous increase in *PHGDH* expression.

**Results:**

Tissues and cells derived from *PHGDH*^*tetO*^ mice exhibit increased serine biosynthesis. Histological examination of skin tissue from *PHGDH*^*tetO*^ mice reveals the presence of melanin granules in early anagen hair follicles, despite the fact that melanin synthesis is closely coupled to the hair follicle cycle and does not normally begin until later in the cycle. This phenotype occurs in the absence of any global change in hair follicle cycle timing. The aberrant presence of melanin early in the hair follicle cycle following PHGDH expression is also accompanied by increased melanocyte abundance in early anagen skin.

**Conclusions:**

These data suggest increased PHGDH expression impacts normal melanocyte biology, but PHGDH expression alone is not sufficient to cause cancer.

**Electronic supplementary material:**

The online version of this article (10.1186/s12885-019-5933-5) contains supplementary material, which is available to authorized users.

## Background

D-3-phosphoglycerate dehydrogenase (PHGDH) is the first enzyme in the de novo serine biosynthesis pathway. Flux through this pathway can be important for the proliferation of some cancer cells, and the *PHGDH* gene is located in a region of focal genomic copy number gain that is associated with subsets of breast cancer and melanoma as well as cell lines derived from other cancer types [1, 2]. *PHGDH*-amplified cells are dependent on expression of catalytically active enzyme to proliferate [3], and high PHGDH expression is associated with negative clinical outcomes in breast cancer [1, 2, 4], glioma [5], cervical cancer [6], lung cancer [7], and colon cancer [8]. In addition to gene amplification, PHGDH expression can be upregulated through transcriptional and epigenetic mechanisms [9–11]. However, whether increased PHGDH expression in tissues promotes cancer initiation or progression and what impact increased enzyme activity has on normal physiology has not been studied.

Because *PHGDH* gene copy number gain is observed with higher frequency in melanoma compared to other cancers [1, 2] and PHGDH expression can accelerate melanoma progression in mice [12], the effect of PHGDH expression on melanocyte biology is of particular interest [13]. Melanocytes are the main pigment-producing cells in mammals. In mice, cutaneous melanocytes in truncal skin are exclusively follicular. Melanogenesis in follicular melanocytes is closely coupled to hair follicle (HF) cycling. Once a HF and the first hair are formed during morphogenesis, the entire base of the HF, the cycling portion, undergoes programmed cell death during a period known as catagen. The HF then enters a “resting” phase, telogen, before the anagen period [14, 15] during which the entire lower portion of the HF is repopulated from epithelial and melanocyte stem cells located in the bulge region [16, 17]. Initiation of melanogenesis is tightly coupled to anagen progression [18], with the first melanin granules typically visible in the HF during the anagen IIIa stage, when the hair follicle bulb extends to the border of the dermis and subcutis [19]. Though serine biosynthesis is not obviously connected to HF cycling, serine biosynthesis pathway enzymes may affect differentiation of survival of stem cells [20, 21], which could potentially perturb HF cycle progression.

To study how increased PHGDH expression affects normal tissue function in mice, we developed a transgenic mouse harboring a human PHGDH cDNA under the control of a doxycycline-inducible promoter. We found that expression of PHGDH results in premature appearance of melanin in HFs as well as an increased number of melanocytes in whole skin, suggesting that PHGDH expression affects melanocyte proliferation and/or differentiation, which may contribute to selection for increased PHGDH expression in cancer.

## Methods

### Generation of *PHGDH*^*tetO*^ mice & mouse husbandry

A previously described system was used to generate *PHGDH*^*tetO*^ mice [22]. Briefly, human PHGDH cDNA with GenBank Accession BC011262.1 from Open Biosystems (MHS1010–73507) was amplified with the following primers:

PHGDH MfeI F: 5′-CAATTGGCCACCATGGCTTTTGCAAATCTGCGGAAAGT-3′.

PHGDH Mfe R: 5′-CAATTGTTAGAAGTGGAACTGGAAGGCTTCAG-3′.

This insert was digested with MfeI from NEB (R0589) and cloned into the EcoRI sites in the pgk-ATG-frt plasmid from Addgene (#20734) to generate a targeting plasmid using standard molecular biology techniques. Sequencing was used to screen for the correct insert orientation and confirm cDNA sequence. The targeting plasmid was co-electroporated with pCAGGS-flpE plasmid (Addgene, # 20733) into F1 C57BL/6 x 129S4 hybrid KH2 ES cells. The KH2 cells as well as pgk-ATG-frt and pCAGGS-flpE-puro were kind gifts from Rudolf Jaenisch (plasmids via Addgene). Clonal selection of ES cells was performed with 150 μg/ml hygromycin B for 9 days, and 8 individual clones were screened by Southern blot as described below. Two ES clones with a properly integrated PHGDH transgene in the Col1a1 locus were injected independently into C57BL/6 blastocysts to produce chimeric mice. The chimeric C57BL/6 x 129S4 *PHGDH*^*tetO*^ transgene founder mice were mated to C57BL/6 background and some showed germline transmission. Before this study and over the course of these experiments, the mice were continually backcrossed onto the C57BL/6 background.

All other mice used for this study were obtained from the Jackson Laboratory with the exception of the iDCT-GFP (01XT4) mice, which were obtained from the NCI Mouse Repository. All animals were housed in the Koch Institute Animal Facility in accordance with the policies of the facility. All animals were housed with at most five individuals per cage, with ad libitum access to food and water. Mice were assigned to experimental groups based on genotypes, and the order in which animals were treated was random. All animals were monitored at least daily and showed no signs of health problems before treatment. All animals continued to be monitored at least daily throughout and after treatment. No adverse events were observed. All mice were euthanized using carbon dioxide according to protocols approved by the MIT Committee on Animal Care.

### Southern blotting

Genomic DNA was digested with SpeI from NEB (R0133). Digested DNA was then separated on an agarose gel, and neutral transfer was performed overnight using Hybond-XL membrane from GE Healthcare Biosciences (RPN303S). Membrane was crosslinked using a Stratalinker UV Crosslinker from Stratagene. The membrane was incubated with Stratagene QuickHyb Hybridization Solution from Agilent (201220). The probe was prepared from the Col1a-3’probe plasmid from Addgene (#20731) by digesting with XbaI and PstI from NEB (R0145 and R0140) and gel purifying the released probe. Purified probe was denatured, then labeled using α-^32^P-dCTP from PerkinElmer Life Sciences (BLU013H) and the Rediprime II DNA Labeling System from GE Healthcare Life Sciences (RPN1633) according to kit instructions. Labeled probe was then purified with Micro Bio-Spin P-6 Gel Columns from Bio-Rad (#732–6200) according to company instructions. Purified, labeled probe was mixed with salmon sperm DNA from Stratagene (201190). Immediately before using, probe was denatured. The probe was then incubated with the membrane and hybridization solution for 1 h at 68 °C. The membrane was washed, then exposed to autoradiography film with an intensifier screen before developing.

### PCR genotyping

PCR genotyping was performed using standard molecular biology techniques using primers and conditions described in the supplementary materials.

### Western blotting

Western blots were performed using standard techniques with primary antibodies against PHGDH (Sigma, HPA021241), β-actin (abcam, ab1801), GAPDH (Cell Signaling Technology, 2118S), Hsp90 (Cell Signaling Technology, #4877), or vinculin (abcam, ab18058) and detected using HRP-conjugated secondary antibodies and chemiluminescence.

### Generation of Embryonic Fibroblasts & Cell Culture

MEFs were prepared from E13.5 *PHGDH*^*tetO/tetO*^, *PHGDH*^*tetO/+*^ or *PHGDH*^*+/+*^ embryos with the *Rosa26-M2rtTA* allele using standard protocols. MEFs were maintained in DMEM with pyruvate (VWR, 10–013-CV), 10% tet-free FBS (VWR Seradigm, Lot 120B14), 2 mM glutamine (Invitrogen, 25030–164), penicillin/streptomycin (VWR, 45000–652) and 3.5 μl beta-mercaptoethanol (Sigma-Aldrich, M3148-25ML) per 500 ml DMEM.

### Mass spectrometry

MEFs were grown in medium supplemented with 1 μg/ml doxycycline for 4 days before extraction. Cells were extracted in ice cold 1:4:5 water:methanol:chloroform with valine-D8 as an internal standard. The aqueous layer was dried under N_2_ and resuspended in 1:1 water:acetonitrile. Samples were analyzed by LC/MS using a QExactive benchtop orbitrap mass spectrometer equipped with a heated electrospray ionization (HESI) probe, coupled to a Dionex UltiMate 3000 UPLC system (Thermo Fisher Scientific, San Jose, CA). Samples were separated by injecting 10 μl of each sample onto a ZIC-pHILIC 2.1 × 150 mm (5 μm particle size) column (EMD). Flow rate was set to 100 μL/min, column compartment was set to 25 °C, and autosampler sample tray was set to 4 °C. Mobile Phase A consisted of 20 mM ammonium carbonate, 0.1% ammonium hydroxide. Mobile Phase B was 100% acetonitrile. The mobile phase gradient (%B) was as follows: 0 min 80%, 5 min 80%, 30 min 20%, 31 min 80%, 42 min 80%. All mobile phase was introduced into the ionization source set with the following parameters: sheath gas = 40, auxiliary gas = 15, sweep gas = 1, spray voltage = − 3.1 kV or + 3.0 kV, capillary temperature = 275 °C, S-lens RF level = 40, probe temperature = 350 °C. Metabolites were monitored using a targeted selected ion monitoring (tSIM) method in negative mode with the quadrupole centered on the M-H ion m + 1.5, m + 2.5, or m + 3.5 mass with a 8 amu isolation window, depending on the number of carbons in the target metabolite. Resolution was set to 70,000, full-scan AGC target was set to 106 ions, and tSIM AGC target was set to 105 ions. Relative quantitation of polar metabolites was performed with XCalibur QuanBrowser 2.2 (Thermo Fisher Scientific) using a 5 ppm mass tolerance and referencing an in-house library of chemical standards. Concentration was normalized to cell number.

### Histology

Tissues were fixed overnight to 24 h in 10% formalin (VWR, 48218–700) and stained with hematoxylin (Leica Biosystems, Wetzlar, Germany, 3801575) and eosin (Leica Biosystems, 3801606) using standard techniques. Slides were scanned using an Aperio slide scanner (Leica Biosystems), and images were analyzed using Aperio ImageScope.

### Hair follicle synchronization and quantitation of melanin in hair follicles

To synchronize HFs, mice were anesthetized and skin was plucked over two 1 cm^2^ areas halfway down the back of mice equidistant from the spine. After the procedure, mice were given carprofen at 3 mg/kg once per day for 3 days as an analgesic. HFs with the bulb located entirely in the dermis were considered to be early anagen. HFs with a lower bulb were not included in the analysis. Only HFs with a fully visible bulb were included in the analysis and all sections were de-identified for blinded quantitation. Each HF assessed for the presence of melanin granules was classified as “none,” “one,” “two” or “three or more”, and each distinct granule was counted as one regardless of size.

### RT-qPCR

RNA was collected from skin using Trizol reagent (Ambion). Skin samples were digested in 1 mL of Trizol using a GentleMACS tissue homogenizer and RNA was isolated according to standard protocol. RNA from FACS samples was isolated using the RNAqueous Micro Kit (Ambion). cDNA was reverse transcribed using an iScript cDNA Synthesis Kit. RT-qPCR was performed with SYBR Green on a LightCycler 480 II machine from Roche. Primers were used at a final concentration of 1 μM and primer sequences are specified in the supplementary materials.

### Flow cytometry

Synchronized skin was dissected from mice, then cut into small pieces in a Petri dish using dissecting scissors. The skin was resuspended in 5 mL sterile PBS with 3 mg/mL dispase II (Roche), 1 mg/mL collagenase I (Worthington Biochemical), and 0.1 mg/mL DNase I (Sigma-Aldrich). This solution was incubated at 37 °C for 30 min, then EDTA was added to a final concentration of 10 mM to stop the digestion reaction. The digested skin was passed through a 70 μm cell strainer then washed twice with sterile PBS. Cells were stained with 1 μg/mL DAPI for 15 min as a live-dead marker, then analyzed for GFP expression on BD FACSAria III flow cytometer. FlowJo software was used to analyze data, which was tested for statistical significance using an unpaired Student’s t test.

## Results

### Generation of a *PHGDH*^*tetO*^ allele

In order to model the consequences of *PHGDH* copy number gain observed in cancer and study the effect of increased PHGDH expression in tissues, a transgenic mouse was engineered to enable tissue-directed PHGDH expression using a previously described system [22]. A human PHGDH cDNA under the control of the tetracycline operator minimal promoter (tetO) was introduced into embryonic stem (ES) cells containing a reverse tetracycline transactivator (*M2rtTA*) allele under the control of the endogenous Rosa26 promoter that is active in most tissues, and these ES cells were used to generate mice with the *PHGDH*^*tetO*^ allele (Additional file 1: Figure S1A-D).

### Characterization of *PHGDH*^*tetO*^ mice

**Fig. 1:**
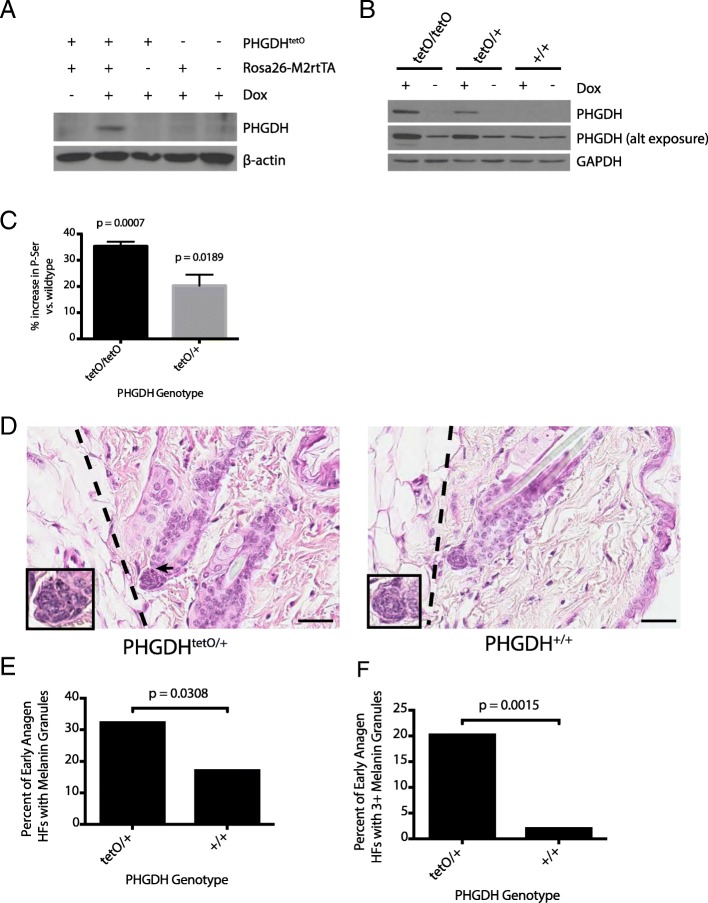
Introducing a *PHGDH*^*tetO*^ allele into mice increases PHGDH expression and results in the aberrant appearance of melanin granules. **a** Western blot analysis to assess PHGDH expression in liver lysates from mice harboring the indicated alleles that were exposed to a doxycycline containing diet (Dox) or a control diet for 5 days. β-actin expression was also assessed as a loading control. **b** Western blot analysis to assess PHGDH expression in MEFs derived from mice with the *Rosa26-M2rtTA* allele and the indicated number of *PHGDH*^*tetO*^ (tetO) alleles that were cultured in media with or without doxycycline (Dox) as for 72 h as indicated. Both a light and dark exposure (alt exposure) are shown, as is GAPDH expression as a loading control. **c** The percent increase in the concentration of intracellular phosphoserine (P-ser) in MEFs derived from mice described in (**b**) harboring one (tetO/+) or two (tetO/tetO) transgene alleles relative to levels found in MEFs derived from wildtype mice is shown. All MEFs were cultured for 4 days in media with doxycycline prior to measurement of P-Ser levels by LC-MS. Data shown represent the mean (+/− SEM). The increase is statistically significant with *p* values from two-tailed Student’s T test. **d** Representative H&E staining of skin sections from 3.5-month-old mice of the indicated genotypes that had been exposed to a doxycycline-containing diet for 9 days. Dotted lines delineate the border between the dermis and the subcutis. Arrow indicates melanin granules in the hair follicles (HFs) of the *PHGDH*^*tetO*^;*Rosa26-M2rtTA* mouse. All hair follicle shown are early anagen as they are contained completely within the dermis. Images were obtained at 40x magnification. Scale bar = 30 μm. Inset images are magnified 80X. **e** Quantitation of the percent of early anagen hair follicles (HF) in each genotype that contain any melanin granules. Data shown represent the % observed when analyzing 167 HFs from one PHGDH^tetO^;*Rosa26-M2rtTA* mouse and 46 HFs from one wild type mouse. **f** Quantitation of the percent of early anagen hair follicles (HF) in each genotype with three or more melanin granules. Data shown represent the % observed when analyzing 167 HFs from one PHGDH^tetO^;*Rosa26-M2rtTA* mouse and 46 HFs from one wild type mouse. The percent increase in hair follicles with melanin granules shown in (**e**) and (**f**) is statistically significant with *p* values derived from one-tailed Fisher’s exact test.

PHGDH is only expressed in tissues from mice with both the *PHGDH*^*tetO*^ and the *Rosa26-M2rtTA* alleles and only upon exposure of tissues to doxycycline (dox) (Fig. 1a). To test whether increased PHGDH expression affects viability, breeding pairs of *PHGDH*^*tetO*^ hemizygotes were continually fed a diet containing dox to induce PHGDH expression in most mouse tissues. Offspring from these crosses were born in expected Mendelian ratios (Additional file 1: Figure S1E). Expression from the Rosa26 promoter is active by the blastocyst stage of the developing embryo [23], and dox readily crosses the placenta to regulate transgene expression in the developing embryo [24–27]. Thus, this result suggests that increased PHGDH expression in the embryo does not prevent mouse development or viability.

Embryonic fibroblasts (MEFs) derived from *PHGDH*^*tetO*^;*Rosa26-M2rtTA* mice display dose-dependent, dox-inducible PHGDH expression (Fig. 1b). The antibody used throughout this study recognizes both human and mouse PHGDH proteins with similar affinities by Western blot (Additional file 1: Figure S1F); thus, PHGDH expression observed in *PHGDH*^*+/+*^ MEFs and in conditions without dox-induced transgene activation reflect mouse PHGDH protein expressed from the endogenous locus. In previous studies examining a variety of cell lines and tissues, expression of PHGDH at the protein level correlates with serine biosynthesis pathway flux [1, 2, 28]. Similar results are obtained following transgene expression, as dox-treated *PHGDH*^*tetO*^;*Rosa26-M2rtTA* MEFs show a dose-dependent increase in both PHGDH protein and the concentration of the unique serine biosynthesis pathway intermediate phosphoserine compared to dox-treated wildtype MEFs (Fig. 1 b-c). These data suggest the transgene expression can increase serine biosynthesis in cells.

### Mice with long-term PHGDH overexpression are grossly normal

*PHGDH*^*tetO*^;*Rosa26-M2rtTA* mice were exposed to dox diet beginning at 6 weeks of age and maintained on this diet for 16–18 months. During this time, mice were monitored weekly without evidence of any obvious abnormalities while alive and at necropsy. Liver and skin samples were analyzed by Western blot for PHGDH protein expression. Some samples showed less PHGDH expression than expected after 16–18 months of dox exposure (Additional file 1: Figure S2), but liver and skin samples from the same individual showed consistent expression levels suggesting that differences in transgene silencing might underlie the variability in expression between mice. Histological analysis of skin, brain, white and brown fat, mammary gland, pancreas, liver, spleen, kidney, colon, lung and heart tissue in this cohort from control mice and *PHGDH*^*tetO*^;*Rosa26-M2rtTA* mice with high PHGDH expression by Western blot was unremarkable, suggesting that mice with long-term increased PHGDH expression are grossly normal.

### Early anagen hair follicles in *PHGDH*^*tetO*^ mice inappropriately contain melanin granules

Given the high frequency of *PHGDH* gene copy number gain observed in melanoma compared to other cancers [1, 2], we sought to examine the effect of PHGDH expression on melanocyte biology. When examining the skin of 3.5 month-old mice treated with dox for 9 days, an anomaly in follicular melanin was observed (Fig. 1d). The bulbs of the HFs pictured in Fig. 1d are surrounded entirely by dermis, identifying them as early anagen, before melanin synthesis has usually begun; however, in *PHGDH*^*tetO*^;*Rosa26-M2rtTA* HFs melanin granules are visible. Ordinarily, during catagen, all cells from the cycling portion of the HF undergo apoptosis, including the melanocytes. Any melanin they have produced is passed to the keratinocytes that make up the hair itself, so that melanin is no longer present in the bulb before new melanin is produced in anagen IIIa of the next HF cycle. Occasionally, melanin granules produced in a previous HF cycle will not be extruded with the hair shaft and are visible in the dermal papilla in telogen, anagen I, or anagen II [29]. However, the *PHGDH*^*tetO*^;*Rosa26-M2rtTA* skin had a significantly greater proportion of early anagen HFs displaying melanin than the wildtype skin (Fig. 1e). Furthermore, although some early anagen HFs in the wildtype skin displayed one or two melanin granules, almost none had three or more (Fig. 1F). In contrast, many melanin-containing follicles in the *PHGDH*^*tetO*^;*Rosa26-M2rtTA* skin had as many as 5–10 granules.

### PHGDH expression does not globally affect timing of the hair follicle cycle

**Fig. 2:**
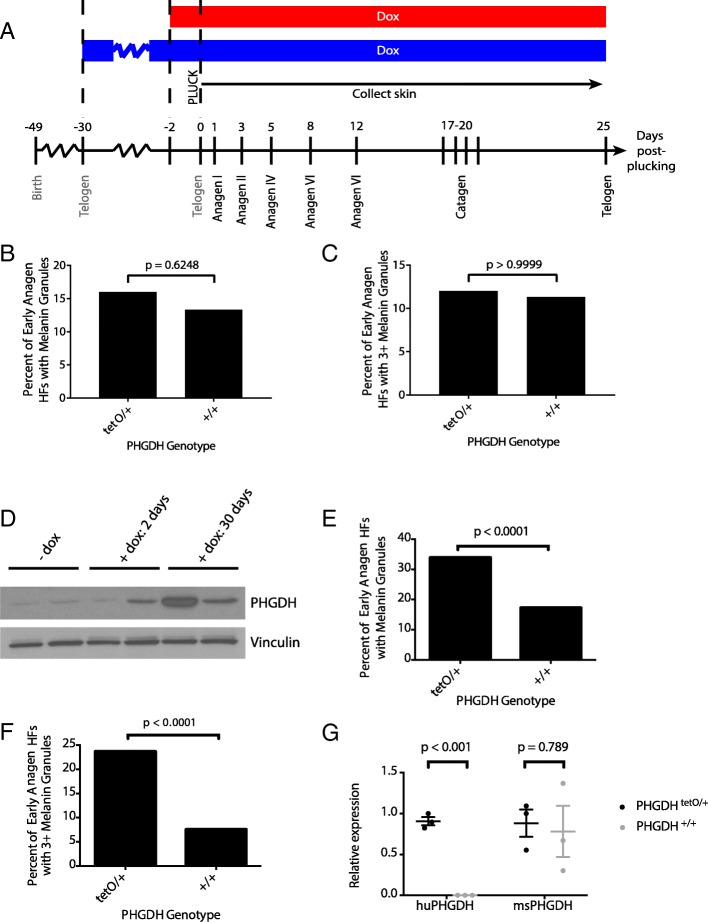
PHGDH expression during the previous hair follicle cycle leads to increased melanin accumulation. **a** A region of hair was plucked from 49 day-old mice (at the second telogen) to synchronize the hair follicle cycle, and skin samples were collected at defined days thereafter. Data were collected from *PHGDH*^*tetO*^;*Rosa26-M2rtTA* (tetO/+) or control (+/+) mice that were exposed to doxycycline (Dox) for either 2 days or 30 days prior to synchronization. Shown is a schematic of the experiment, with the red bar depicting mice exposed to doxycycline diet for 2-days before synchronization, and the blue bar depicting mice exposed to doxycycline for 30-days before synchronization. **b** Quantitation of the percent of early anagen hair follicles (HFs) containing any melanin granules in *PHGDH*^*tetO*^;*Rosa26-M2rtTA* (tetO/+) or control (+/+) mice exposed to doxycycline for 2 days prior to synchronization. Data shown represent the % observed when analyzing 50 HFs per mouse from 3 mice of each genotype (**c**) Quantitation of the percent of early anagen hair follicles with three or more melanin granules in *PHGDH*^*tetO*^;*Rosa26-M2rtTA* (tetO/+) or control (+/+) mice exposed to doxycycline for 2 days prior to synchronization. Data shown represent the % observed when analyzing 50 HFs per mouse from 3 mice of each genotype. No statistically significant increase in hair follicles with melanin granules were observed in (**b**) or (**c**) with *p*-values derived from two-tailed Fisher’s exact test. **d** Western blot analysis for PHGDH expression in skin from *PHGDH*^*tetO*^;*Rosa26-M2rtTA* mice never exposed to doxycycline-containing diet (−dox) or fed a doxycycline-containing diet for 2 or 30 days as indicated. Vinculin expression is also shown as a loading control. **e** Quantitation of the percent of early anagen hair follicles (HFs) containing any melanin granules in *PHGDH*^*tetO*^;*Rosa26-M2rtTA* (tetO/+) or control (+/+) exposed to doxycycline for 30 days prior to synchronization. Data shown represent the % observed when analyzing 50 HFs per mouse from 3 mice of each genotype. **f** Quantitation of the percent of early anagen hair follicles with three or more melanin granules *PHGDH*^*tetO*^;*Rosa26-M2rtTA* (tetO/+) or control (+/+) mice exposed to doxycycline for 30-days prior to synchronization. Data shown represent the % observed when analyzing 50 HFs per mouse from 3 mice of each genotype. The increase in hair follicles with melanin granules shown in (**e**) and (**f**) is statistically significant with *p*-values derived from two-tailed Fisher’s exact test. **g** qPCR to assess species-specific PHGDH expression in early anagen skin isolated from *PHGDH*^*tetO*^;*Rosa26-M2rtTA* (tetO/+) or control (+/+) mice exposed to doxycycline for 30-days prior to synchronization. An increase in human PHGDH (huPHGDH), but not mouse PHGDH (msPHGDH) expression is statistically significant with p values derived from unpaired Student’s t test. Data shown represent the mean (+/− SD)

To further characterize this phenotype, HF cycling was synchronized by plucking hair from a region of skin to induce HFs in that region to enter a new cycle. Skin was then harvested at defined time points to examine a desired cycle stage [19]. In order to determine the effect of PHGDH overexpression on follicular melanin throughout the HF cycle, two approaches were used: one set of mice was fed dox diet for 2 days before plucking (red bar) and the other set for 30 days before the HF cycle was synchronized by plucking (blue bar) (Fig. 2a). The first few HF cycles following birth are relatively synchronous across individuals [19]. Therefore, a 30-day pre-induction with dox followed by plucking at 49 days of age allows PHGDH overexpression during the entire cycle preceding plucking, from telogen to telogen. Conversely, the 2-day pre-induction only allowed PHGDH overexpression during the very end of the HF cycle preceding synchronization. By using two different pre-induction times, we aimed to determine whether the melanin phenotype required PHGDH overexpression in only the current HF cycle or if expression in the preceding cycle was required for melanin accumulation.

Examination of skin at various time points after HF synchronization in mice exposed to dox for 30 days prior to plucking suggested PHGDH overexpression does not globally affect timing of the HF cycle (Additional file 1: Figure S3A). HFs in synchronized skin from both control and *PHGDH*^*tetO*^;*Rosa26-M2rtTA* mice were found in the expected stages for their collection days. Additionally, no perceptible differences visible by H&E staining were evident in any HF stage other than early anagen. The fact that the phenotype is early anagen-specific likely explains why it was not detected in the cohort of aged mice. Early anagen is relatively short compared to the entire HF cycle; thus, early anagen HFs are not abundant in mice of any age. Furthermore, HF cycling becomes more asynchronous as mice age [30], so that the likelihood of collecting a skin sample by chance with an abundance of early anagen HFs is further decreased.

### Early anagen hair follicles in synchronized *PHGDH*^*tetO*^ skin contain melanin granules

Early anagen follicles in synchronized skin of *PHGDH*^*tetO*^;*Rosa26-M2rtTA* mice with only a 2-day dox pre-induction show the presence of melanin granules, but neither the proportion of HFs with melanin (Fig. 2b) nor the fraction of HFs with three or more melanin granules (Fig. 2c) is significantly different than in wild-type mice. Analyzing skin by Western blot shows that 2-day pre-induction is sufficient to moderately increase PHGDH levels in some mice; however, the change in expression is higher in the skin of mice exposed to dox diet for 30 days (Fig. 2d), raising the possibility that the absence of a melanin phenotype after 2 days of dox pre-induction is due to the latency of PHGDH expression.

Examination of early anagen skin from mice with a 30-day pre-induction showed melanin accumulation in *PHGDH*^*tetO*^;*Rosa26-M2rtTA* mice to a degree that reproduced the initial phenotype observed (Additional file 1: Figure S3B). The early anagen HFs from the skin of *PHGDH*^*tetO*^;*Rosa26-M2rtTA* mice more frequently contained melanin (Fig. 2e) and were more likely to have a high number of melanin granules (Fig. 2f) than their wild-type counterparts. The percentages observed in this experiment were similar to those observed in the initial unsynchronized experiment (Fig. 1d-f). To confirm that melanin accumulation is associated with increased PHGDH expression from the transgene, we designed qPCR primers specific for human or mouse PHGDH cDNA (Additional file 1: Figure S4A-D) and found that in synchronized *PHGDH*^*tetO*^;*Rosa26-M2rtTA* skin, expression of human PHGDH was increased while expression of mouse PHGDH was unchanged (Fig. 2g).

### Melanin accumulation in *PHGDH*^*tetO*^ mice is caused by cell autonomous PHGDH expression and is not dependent on PHGDH overexpression in catagen

**Fig. 3:**
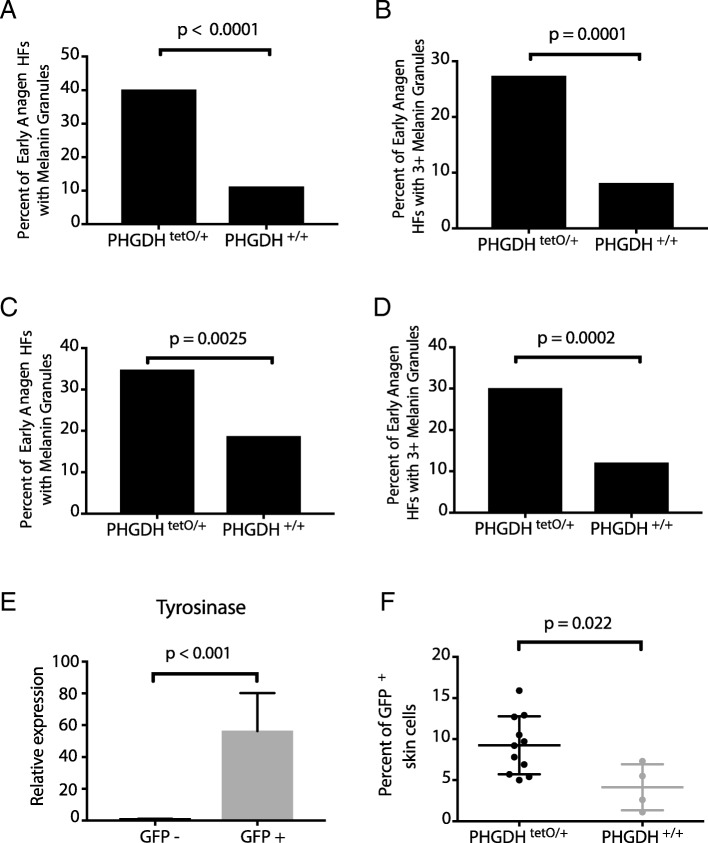
Increased PHGDH expression in melanocytes drives melanin accumulation in early anagen hair follicles and increases melanocyte abundance. **a** *PHGDH*^*tetO*^ mice were crossed to *Dct-rtTA* mice to drive increased PHGDH expression solely in melanocytes. Quantitation of the percent of early anagen hair follicles (HFs) containing any melanin granules in skin from *Dct-rtTA* mice with the indicated *PHGDH*^*tetO*^ genotype exposed to doxycycline for 30 days prior to hair follicle synchronization as described in Fig. 2. Data shown represent the % observed when analyzing 50 HFs per mouse from 3 mice of each genotype. **b** Quantitation of the percent of early anagen hair follicles (HFs) with three or more melanin granules in in skin from mice described in (**a**) exposed to doxycycline for 30 days prior to hair follicle synchronization. Data shown represent the % observed when analyzing 50 HFs per mouse from 3 mice of each genotype. **c** Quantitation of the percent of early anagen hair follicles (HFs) containing any melanin granules in skin from mice described in (**a**) exposed to doxycycline for 2 days prior to hair follicle synchronization. Data shown represent the % observed when analyzing 50 HFs per mouse from 3 mice of each genotype. **d** Quantitation of the percent of early anagen hair follicles (HFs) with three or more melanin granules in skin from mice described in (**a**) exposed to doxycycline for 2 days prior to hair follicle synchronization. Data shown represent the % observed when analyzing 50 HFs per mouse from 3 mice of each genotype. The increase in hair follicles with melanin granules shown in (**a**-**d**) is statistically significant with p-values derived from two-tailed Fisher’s exact test. **e** *PHGDH*^*tetO*^; *Dct-rtTA* mice were crossed to *H2B-GFP*^*tetO*^ mice such that melanocytes would express both PHGDH and GFP. qPCR to assess tyrosinase expression (a melanocyte-specific enzyme) in GFP- and GFP+ cells isolated from *PHGDH*^*tetO*^*;Dct-rtTA; H2B-GFP*^*tetO*^ mice exposed to doxycycline for 30 days prior to hair follicle synchronization. Data shown represent the mean (+/− SD). The increase in tyrosinase expression is significant with p-values derived from an unpaired Student’s t test. **f** Mice described in (**e**) with or without a *PHGDH*^*tetO*^ allele we exposed to doxycycline for 30-days prior to hair follicle synchronization and subsequent collection of early anagen skin samples. Cells isolated from skin were analyzed by flow cytometry to assess GFP+ melanocyte abundance. Data shown represent the mean (+/− SD). The increase in GFP+ melanocytes from PHGDH^tetO/+^ mice is statistically significant with p-values derived from an unpaired Student’s t test

In order to determine whether the melanin phenotype observed in the *PHGDH*^*tetO*^;*Rosa26-M2rtTA* mice is cell autonomous, we crossed *PHGDH*^*tetO*^ mice to mice harboring a *Dct-rtTA* allele that allows melanocyte-specific transgene expression [31, 32]. With a 30-day pre-induction, skin from *PHGDH*^*tetO*^;*Dct-rtTA* mice displayed melanin granules in early anagen HFs with similar percentages as those observed in mice with a *Rosa26-M2rtTA* allele (Fig. 3a-b), suggesting that the PHGDH-dependent presence of melanin in early anagen HFs is a melanocyte-autonomous event.

To evaluate whether PHGDH expression is required in the previous HF cycle for this phenotype, we used a 2-day pre-induction with dox. Though a 2-day pre-induction led to only weak PHGDH expression when driven by *Rosa26-M2rtTA*, the melanocyte specific *Dct-rtTA* is predicted to promote higher PHGDH expression in these cells. Indeed, we found that with a 2 day pre-induction, skin from *PHGDH*^*tetO*^;*Dct-rtTA* mice displayed melanin granules in early anagen HFs at a higher rate than skin from wildtype mice (Fig. 3c-d). The presence of the melanin phenotype with a 2-day pre-induction suggests that the phenotype does not depend on PHGDH overexpression during the previous catagen. This argues against PHGDH promoting survival of melanocytes that would normally die during the previous catagen phase. Instead, the effect of PHGDH expression on uncoupling melanin appearance with normal HF cycle progression only requires the presence of PHGDH during the earliest phases of the HF cycle.

### Increased PHGDH expression in melanocytes increases melanocyte abundance in early anagen skin

To determine whether the presence of excess melanin granules in early anagen HFs is related to a change in melanocyte number, we quantified melanocyte abundance using flow cytometry. To quantify melanocytes, *PHGDH*^*tetO*^; *Dct-rtTA* mice were crossed to *H2B-GFP*^*tetO*^ mice [31–33] so that melanocytes would express both PHGDH and GFP. The resulting mice were then exposed to a dox diet for 30 days, plucked, and skin was collected in early anagen. Adapting previously described protocols [31, 34], this skin was then dissociated into single-cell suspension and sorted by flow cytometry into GFP-positive and GFP-negative populations in order to quantitate the effect of PHGDH expression on the relative abundance of GFP-positive melanocytes (Additional file 1: Figure S5A). In order to validate that the GFP-positive cells were indeed melanocytes, we performed qPCR for tyrosinase, a melanocyte marker, which was present in GFP-positive cells, and nearly undetectable in GFP-negative cells (Fig. 3e). Conversely, expression of KPRP, a keratinocyte marker, and AdipoQ, an adipocyte marker, were restricted to the GFP-negative cells (Additional file 1: Figure S5B-C). The proportion of GFP-positive cells was significantly higher in early anagen skin from mice with the *PHGDH*^*tetO*^;*Rosa26-M2rtTA* alleles (Fig. 3f), suggesting that melanocytes are more abundant in early anagen skin when PHGDH is overexpressed.

## Discussion

High *PHGDH* expression is observed in select cancer cells and in some cases is necessary for proliferation and survival [1, 2]. Genomic copy number gain involving *PHGDH* is observed with higher frequency in melanoma than in other cancers [1], and in this regard it is interesting that increased PHGDH expression driven by a ubiquitous promoter in mice results in a phenotype involving melanocytes such that progression of the hair follicle cycle is uncoupled from melanin appearance. This effect on normal melanocyte biology may provide insight into how PHGDH expression contributes to melanoma.

A key unanswered question is why hair follicles in *PHGDH*^*tetO*^ mice display an increased number of melanin granules and melanocytes early in the HF cycle. Melanogenesis involves the production, survival and differentiation of melanocytes [35], functional melanosome biogenesis [36], appropriate transcription, translation, modification, and activity of synthetic enzymes such as tyrosinase [37, 38], input from autocrine and paracrine signals [39], and availability of substrate and appropriate chemical conditions for melanogenesis, including pH and redox state [40, 41]. Increased serine synthesis might affect one or more of these processes in melanocytes or melanocyte stem cells. Alterations in redox state may be relevant to PHGDH expression, as increased serine synthesis is associated with resistance to oxidative stress in melanoma and breast cancer [21, 42]. PHGDH could also affect follicular melanogenesis by promoting inappropriate differentiation of melanocytic stem cells into melanocytes or increased melanocyte proliferation. However, the methods used in this work to examine the presence of melanin are insufficient to address whether PHGDH expression alters new melanin synthesis. Given the observation that PHGDH expression is only required for 2 days in order to observe melanin accumulation, it is unlikely that PHGDH expression causing retention of melanin from the prior HF cycles can completely explain the phenotype. Instead, PHGDH expression may impact other processes that lead to the appearance of melanin granules in early anagen HFs. Regardless of the mechanism resulting in aberrant melanin accumulation in hair follicles when PHGDH is expressed, this data illustrates that PHGDH can impact normal melanocyte biology and may contribute to why this gene is amplified in melanoma.

## Conclusions

Long-term expression of PHGDH based on ubiquitous Rosa26-rtTA expression revealed no gross phenotype save an increase in melanin granules in early anagen hair follicles. Importantly, the lack of tumor formation argues that PHGDH expression alone is not sufficient to drive cancer in melanocytes. However, although it is relatively ubiquitous in expression, the *Rosa26-M2rtTA* allele does not drive expression to high levels in all tissues [23]. It is possible that crossing the *PHGDH*^*tetO*^ mouse to other tissue-specific rtTA alleles that drive higher PHGDH expression may lead to additional phenotypes in those tissues.

## Additional file

Additional file 1: **Figure S1.** Generation of the *PHGDH*^*tetO*^ allele. **Figure S2.** Tissues from mice with long-term exposure to doxycycline diet show variable PHGDH expression. **Figure S3.** PHGDH expression leads to melanin accumulation in early anagen hair follicles but does not globally affect timing of the hair follicle cycle. **Figure S4.** Validation of species-specific PHGDH qPCR primers. **Figure S5.** Evidence that adipocytes and keratinocytes sort into the GFP- fraction when cells are isolated from the skin of *PHGDH*^*tetO*^; *Dct-rtTA*; *H2B-GFP*^*tetO*^ mice. **Supplementary methods.** (ZIP 1160 kb)

## Data Availability

The datasets used and/or analyzed during the current study are available from the corresponding author upon reasonable request.

## Abbreviations

dox: Doxycycline
ES cell: Embryonic stem cell
HF: Hair follicle
M2rtTA: Reverse tetracycline transactivator
MEF: Mouse embryonic fibroblast
PHGDH: Phosphoglycerate dehydrogenase
tetO: Tetracycline operator minimal promoter
