# Supplementary material for: Increased PHGDH expression promotes aberrant melanin accumulation
Source: BMC Cancer. 2019 Jul 22;19:723. doi: 10.1186/s12885-019-5933-5 (PMC6647269; doi:10.1186/s12885-019-5933-5)
Supplement: Supplementary file 1 — Figure S1. Generation of the PHGDHtetO allele. Figure S2. Tissues from mice with long-term exposure to doxycycline diet show variable PHGDH expression. Figure S3. PHGDH expression leads to melanin accumulation in early anagen hair follicles but does not globally affect timing of the hair follicle cycle. Figure S4. Validation of species-specific PHGDH qPCR primers. Figure S5. Evidence that adipocytes and keratinocytes sort into the GFP- fraction when cells are isolated from the skin of PHGDHtetO; Dct-rtTA; H2B-GFPtetO mice. Supplementary methods. (ZIP 1160 kb) [file 12885_2019_5933_MOESM1_ESM.zip › Mattaini et al supplementary materialsR2 corrected.docx]

## SUPPLEMENTARY MATERIALS

### Supplementary Methods

PCR genotyping was performed using standard molecular biology techniques using the primers and conditions specified below.

| Allele | Primers | Annealing temp., °C | Wild type allele band size, bp | Transgene allele band size, bp |
| --- | --- | --- | --- | --- |
| *PHGDH^tetO^* | oIMR6724:  5′- CCCTCCATGTGTGACCAAGG -3’  oIMR6725:  5′- GCACAGCATTGCGGACATGC -3’  oIMR6726:  5′- GCAGAAGCGCGGCCGTCTGG -3’  (Sequences from Jackson Laboratory) | 65 | 295 | 493 |
| *Rosa26-M2rtTA* | oIMR8545:  5′-AAAGTCGCTCTGAGTTGTTAT-3’  oIMR8546:  5′-GGAGCGGGAGAAATGGATATG-3’  oIMR8052:  5′-GCGAAGAGTTTGTCCTCAACC-3’  (Sequences from Jackson Laboratory) | 65 | ~650 | 340 |
| *Dct-rtTA* | Y104:  5'-ACTAAGTAAGGATCAATTCAG -3'  Y105:  5'-TGTACTAGGCAGACTGTG-3'  (Sequences from NCI Mouse Repository) | 55 | (No band) | 370 |
| *H2B-GFP* | Y106:  5'-GCCACAAGTTCAGCGTGTCC-3'  Y107:  5'-GATGCCCTTCAGCTCGATGC-3'  (Sequences from NCI Mouse Repository) | 60 | (No band) | 314 |

RT-qPCR was performed using standard molecular biology techniques using the primers specified below.

| Target | Forward Primer | Reverse Primer |
| --- | --- | --- |
| Human PHGDH | 5’-CTGCGGAAAGTGCTCATCAGT-3’ | 5’-TGGCAGAGCGAACAATAAGGC-3’ |
| Mouse PHGDH | 5’-ATGGCCTTCGCAAATCTGC-3’ | 5’-AGTTCAGCTATCAGCTCCTCC-3’ |
| Tyrosinase | 5’-CACCATGCTTTTGTGGACAG-3’ | 5’-GGCTTCTGGGTAAACTTCCAA-3’ |
| AdipoQ | 5’-TGTTCCTCTTAATCCTGCCCA-3’ | 5’-CCAACCTGCACAAGTTCCCTT-3’ |
| KPRP | 5’-AACCCGTTCGTTGTCCCAG-3’ | 5’-TTGGGTGAAGTTATATGAGCCAC-3’ |
| F4/80 | 5’-TGACTCACCTTGTGGTCCTAA-3’ | 5’-CTTCCCAGAATCCAGTCTTTCC-3’ |
| GAPDH | 5’-TGTAGACCATGTAGTTGAGGTCA-3’ | 5’-AGGTCGGTGTGAACGGATTTG-3’ |

### Figure S1. Generation of the *PHGDH^tetO^* allele

**(A)** Schematic of the Col1A locus in wildtype mouse cells (top), the modified locus in KH2 ES cells (middle) and the locus after targeting to introduce the *PHGDH^tetO^* allele (bottom). The PHGDH cDNA introduced into the Col1A locus is the human sequence. The expected band sizes when the indicated probe is used for Southern blot analysis as in **(B)** and **(C)**] are indicated. Also shown are the location of the SpeI sites (marked “S”) in each locus used to digest genomic DNA for Southern blot analysis. FRT, flippase recognition target site; tetO, tetracycline operator minimal promoter. **(B)** Southern blot analysis of SpeI-digested genomic DNA from six *PHGDH^tetO^*-targeted ES cells. Clones D4 and D5 exhibit proper targeting of the Col1A locus and an unaffected wild-type allele. **(C)** Southern blot analysis of SpeI-digested genomic DNA from mice of the indicated genotypes. **(D)** PCR-based genotyping of the *PHGDH^tetO^* and *Rosa26-M2rtTA* alleles. In the *PHGDH^tetO^* reaction, the presence of the transgene is indicated by the upper band; in the *Rosa26-M2rtTA* reaction, the presence of the transgene is indicated by the lower band. **(E)** The number of offspring of each genotype observed when mice hemizygous for the *PHGDH^tetO^* allele exposed to a doxycycline diet were mated. The observed distribution of genotypes in the offspring did not differ significantly from expected Mendelian ratios, with p=0.58 by the χ^2^ goodness-of-fit test. **(F)** Western blot analysis of PHGDH protein using the indicated amount of recombinant human or mouse PHGDH to test antibody specificity.

### Figure S2. Tissues from mice with long-term exposure to doxycycline diet show variable PHGDH expression.

Western blot analysis for PHGDH expression in liver and skin from *PHGDH^tetO^;Rosa26-M2rtTA* (tetO/+) and wildtype (+/+) mice that were exposed to doxycycline diet for 16-18 months. Hsp90 expression is also shown as a loading control.

### Figure S3. PHGDH expression leads to melanin accumulation in early anagen hair follicles but does not globally affect timing of the hair follicle cycle.

**(A)** A region of hair was plucked from 49 day-old mice (at the second telogen) to synchronize the hair follicle cycle, and skin samples were collected at defined days thereafter. Data were collected from *PHGDH^tetO^;Rosa26-M2rtTA* or control (+/+) mice that were exposed to doxycycline (Dox) for 30 days prior to synchronization. Representative H&E staining of skin sections from mice of the indicated genotypes is shown. Images were obtained at 4x magnification. Scale bar = 1 mm. **(B)** Representative H&E staining of skin from a control (PHGDH^+/+^) and *PHGDH^tetO^;R26-M2rtTA* (PHGDH^tetO/+^) mouse showing early anagen hair follicles (HFs) that contain zero and multiple melanin granules, respectively. Images were obtained at 40x magnification. Scale bar = 30 µm.

### Figure S4. Validation of species-specific PHGDH qPCR primers

qPCR primers specific to **(A)** human (hu PHGDH) and**(B)** mouse (ms PHGDH) PHGDH were tested for linearity and relative quantitation compared to 18S rRNA. The slope of each line does not significantly differ from 0, with p-values derived from an F test. The same human **(C)** and mouse **(D)** PHGDH primers were examined for an ability to amplify PHGDH cDNA derived from mouse liver or the human cell line MDA-MB-468 infected with a control shRNA (shRenilla) or one of two hairpins targeting PHGDH (shPHGDH).

### Figure S5. Evidence that adipocytes and keratinocytes sort into the GFP- fraction when cells are isolated from the skin of *PHGDH^tetO^*; *Dct-rtTA*; *H2B-GFP^tetO^* mice.

**(A)** *PHGDH^tetO^*; *Dct-rtTA*; *H2B-GFP^tetO^* mice with melanocytes that express both PHGDH and GFP, along with control mice with melanocytes that express GFP were exposed to doxycycline for 30 days prior to hair follicle synchronization. Early anagen skin samples were isolated, and GFP+ and GFP- cells were isolated via FACS. Cells were gated on the single cell, live (DAPI-) population. GFP+ cells were identified based on signal in a GFP channel compared to autofluorescence as measured by signal in a PE channel using a skin sample from a mouse that did not contain *H2B-GFP^tetO^* (GFP negative) as a negative control. Representative FACS plots from a PHGDH^tetO^ mouse and a wildtype (PHGDH^+/+^) mouse are shown. **(B)** qPCR analysis of cDNA isolated from unsorted, GFP-, and GFP+ cells isolated from the skin of *PHGDH^tetO^*; *Dct-rtTA*; *H2B-GFP^tetO^* mice described in Figure 4E. Primers were used to amplify the keratinocyte-specific gene *KPRP*, and **(C)** the adipocyte-specific gene *AdipoQ* as indicated. Data shown represent the mean (+ SD).
